# Lower Extremity Staged Revascularization (LESR) as a new innovative concept for lower extremity salvage in acute popliteal artery injuries: a hypothesis

**DOI:** 10.1186/s13037-022-00349-2

**Published:** 2022-12-15

**Authors:** Joseph Edwards, Rebecca N. Treffalls, Hossam Abdou, David P. Stonko, Patrick F. Walker, Jonathan J. Morrison

**Affiliations:** 1 R Adams Cowley Shock Trauma Center, University of Maryland, Baltimore, MD USA; 2 Department of Surgery, Johns Hopkins Hospital, Baltimore, MD USA; 3 Department of Vascular and Endovascular Surgery, Mayo Clinic, 200 First Street SW, Rochester, MN 55905 USA

**Keywords:** Limb salvage, Popliteal artery injury, Extracorporeal circuit, Shunt, Ischemia

## Abstract

Popliteal artery injury following knee dislocation is associated with significant morbidity and high amputation rates. The complex and multi-disciplinary input required to manage this injury effectively can take time to arrange, prolonging the time to revascularization. Furthermore, open surgical bypass or interposition graft can be technically challenging in the acute setting, further prolonging ischemic time.

Temporary intravascular shunts can be used to temporarily restore flow but require surgical exposure which takes time. Endovascular techniques can decrease the time to revascularization; however, endovascular popliteal stent-grafting is controversial because the biomechanical forces relating to flexion and extension of the knee may increase the risk of stent thrombosis. An ideal operation would result in rapid revascularization, eventually leading to a definitive and durable surgical solution.

We hypothesize that a staged approach combing extracorporeal shunting, temporary endovascular covered stent placement, external fixation of bony injury, and definitive open repair provides for a superior approach to popliteal artery injury than current standard of care. We term this approach lower extremity staged revascularization (LESR) and the aim is to minimize the known factors contributing to poor outcomes after traumatic popliteal artery injury.

## Introduction

Popliteal artery injury following a traumatic knee dislocation or lower extremity fracture is a challenging clinical scenario, often resulting in an amputation rate of 25% in a civilian setting [1], which has remained unchanged for 30 years [2–4]. Patients with this injury require prompt recognition for revascularization and boney stabilization, which involves the coordination of multiple specialties. The multi-disciplinary treatment for this injury takes time to organize and can prolong the time to revascularization. There is a need to change this paradigm of care to improve patient outcomes.

Endovascular popliteal stent-grafting (EPSG) has been described for popliteal lesions and can be more expeditious than surgical exploration of the popliteal fossa. However, the durability of EPSG has been questioned as spanning the knee subjects the stent to significant biomechanical forces that can lead to ESPG occlusion over time [5]. Prompt revascularization of popliteal artery injuries is critical, with the ultimate need of a durable and definitive vascular reconstruction.

## Hypothesis

**Fig. 1:**
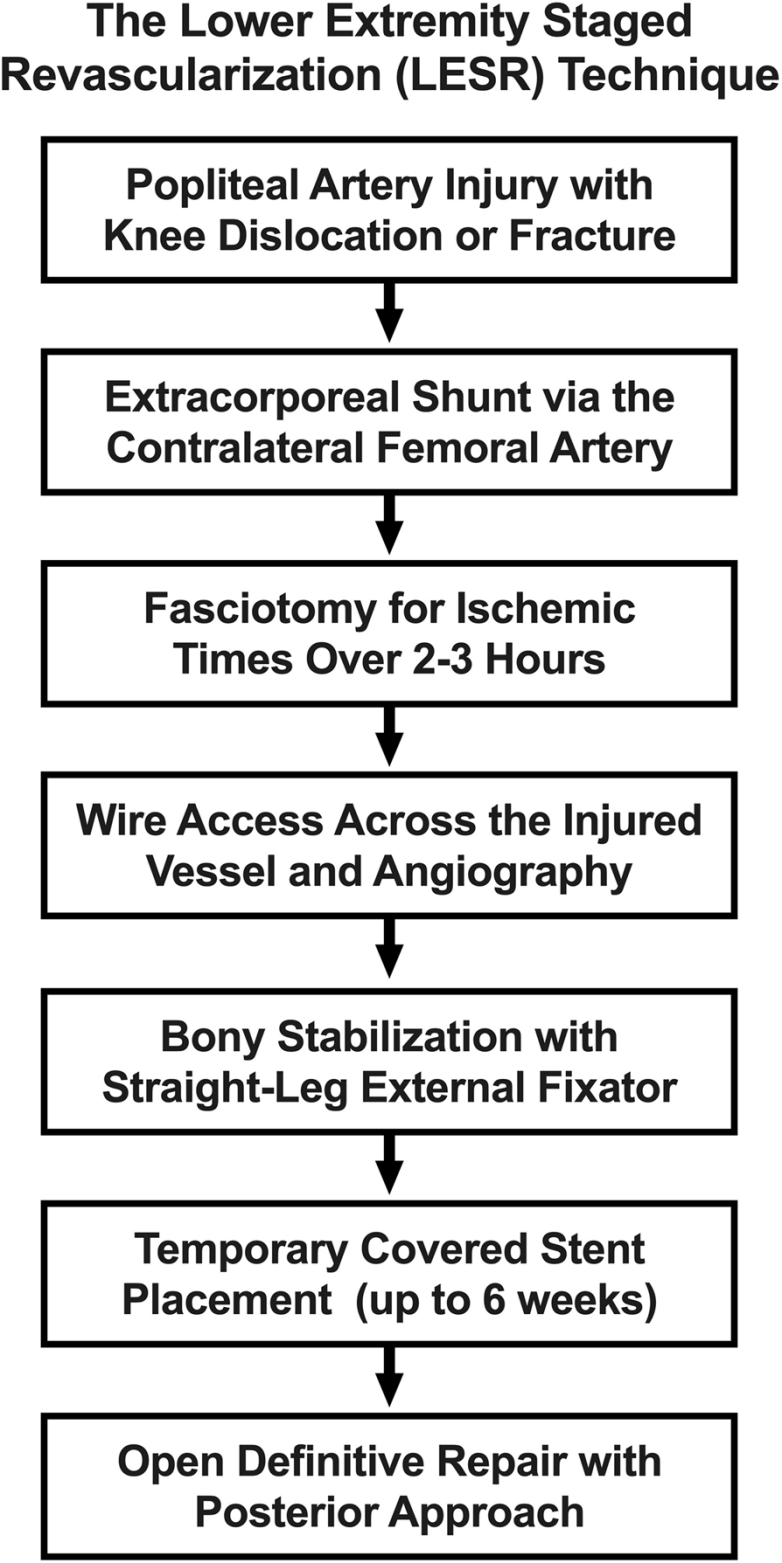
The steps of the lower extremity staged revascularization (LESR) technique.The main steps of LESR are extracorporeal shunting, endovascular damage control, and open definitive repair

We hypothesize that this can be achieved via the lower extremity staged revascularization (LESR) technique, which is a novel staged approach that combines the relative strengths of extracorporeal, endovascular, and open approaches. This article presents the technical steps required to test this hypothesis. The LESR approach consists of three phases: extracorporeal, endovascular damage control, and open reconstruction. Figure 1 details the staged approach.

## The LESR technique

### Extracorporeal shunting

**Fig. 2:**
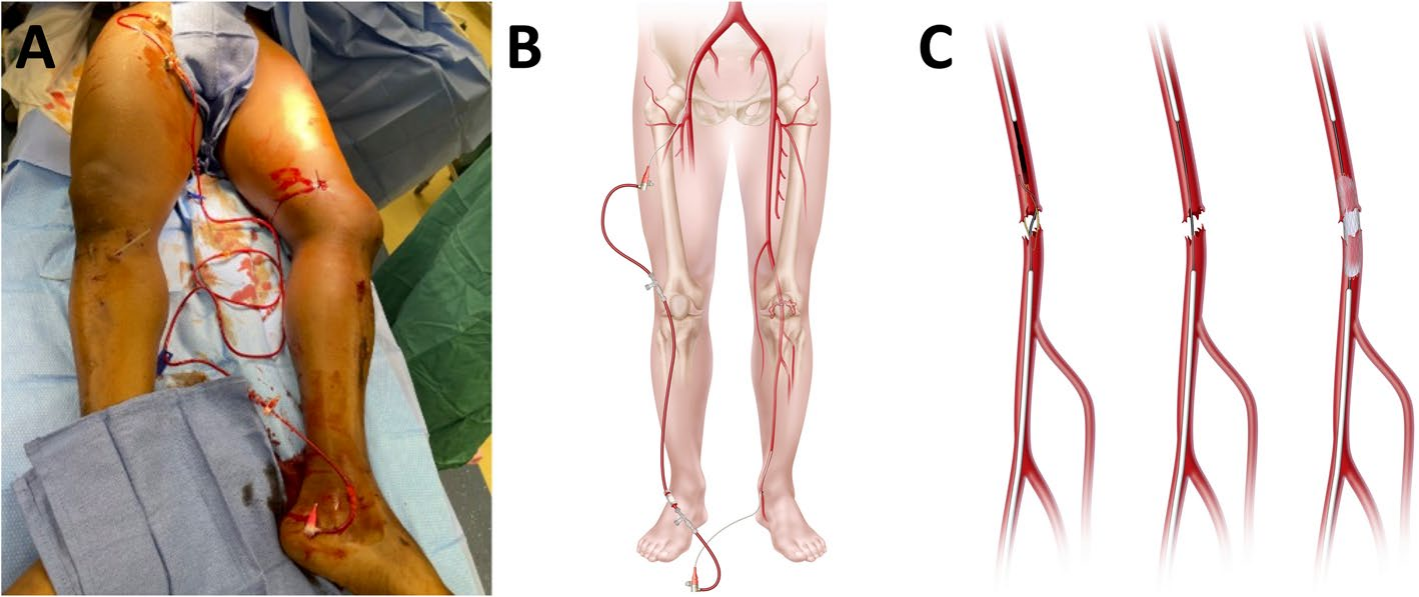
**A** Demonstration of an extracorporeal shunt (exoshunt) in a human patient, **B** Illustration of an exoshunt for lower extremity injury revascularization, and **C** Illustration of lower extremity revascularization using an endovascular approach with retrograde and antegrade vascular access

An extracorporeal shunt (i.e., exoshunt) is an extracorporeal perfusion technique delivered percutaneously at the bedside to minimize warm ischemic time. Retrograde perfusion is performed by placing an arterial access sheath in the contralateral common femoral artery that is then connected via intravenous tubing to a sheath placed in a distal artery of the injured extremity. This use of extracorporeal retrograde flow to an injured extremity limits ischemic time. The access sheaths may also be used intraoperatively for imaging and intervention and ultimately aid in the repair of the injury (Fig. 2). A similar mechanism of exoshunting has been described in a large animal model [6, 7]. However, the technique described by Edwards et al. and Treffalls et al. uses an active pump to increase flow [6, 7], whereas the LESR technique utilizes passive flow.

### Endovascular damage control

#### Fasciotomy

For patients with ischemic time over 2–3 h or with concomitant tibial fracture, strong consideration should be given to early fasciotomy to decrease compartment pressures and optimize distal perfusion. This step allows for a direct assessment of the muscle to help assess whether a limb is salvageable. Thus, as the patient has perfusion maintained by the exoshunt, the operating room can be prepared for fasciotomy.

#### Angiography and wire access

**Fig. 3:**
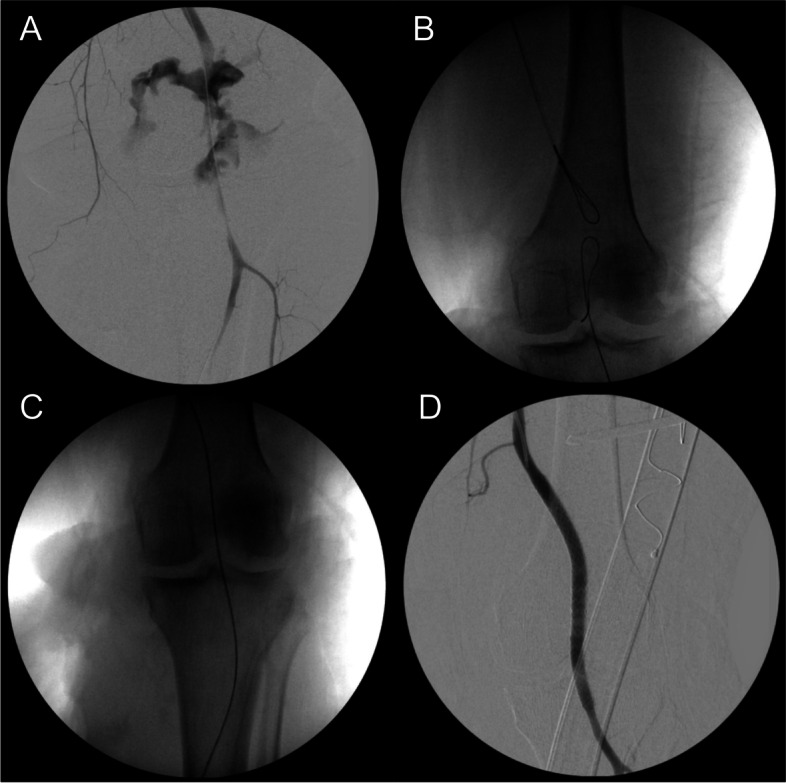
Radiographs of **A** popliteal artery injury, **B** utilization of both antegrade and retrograde vascular access, **C** successful placement of a wire across the injury, and **D** completion angiogram after covered stent placement

The arterial access sheaths are used to facilitate antegrade and retrograde angiography, which helps delineate the anatomy of the injury. A wire is then used to cross the injured vessel, which can be performed in three ways: antegrade, retrograde, or rendezvous access via snare technique (Figs. 2 and 3). This is the critical step in LESR, as endovascular revascularization cannot be attempted without wire access. Heparinization should be performed if the patient does not have a contraindicated concomitant injury.

#### Bony stabilization

The application of a straight-leg external fixator is important for two reasons. First, it stabilizes the fracture or dislocation, reducing further tissue injury. Second, the external fixation maintains the popliteal artery in a straight position to ensure it is receptive to an endovascular stent graft for initial revascularization.

#### Endovascular revascularization

Covered stent placement can be performed by utilizing the antegrade or retrograde technique. This endovascular shunt (i.e., endoshunt) replaces the exoshunt and perfuses the extremity distal to the injury to provide in-line flow. The stent should ideally be landed in the distal popliteal, 2–3 cm from the take-off of the anterior tibial artery. Once the stent has been placed, a completion angiogram should be performed. Other endovascular tools can be considered as required, such as suction thrombectomy, thrombolytic therapy, or intra-arterial nitroglycerin administration to reduce vasospasms.

### Open definitive repair

The endoshunt can be left in place with the lower extremity fixed in a straight position for up to 6 weeks to allow the acute phase of injury to resolve. Antiplatelet therapy should be administered as indicated for the management of a covered stent. A planned open definitive repair can then be performed with a posterior approach preferred. The preservation of a distal cuff of native artery is important so that the stent-graft explant can be performed easily and the distal anastomosis fashioned in a technically efficient manner. Although not optimal in the acute setting, given the need to prone the patient, this approach minimizes disruption to the soft tissue envelope around the knee joint to maximize functional outcomes. This approach also maintains the blood supply to the medial head of the gastrocnemius.

## Implications of the hypothesis

We propose a novel approach to traumatic lower extremity injury using the lower extremity staged revascularization technique to improve limb perfusion and salvage. With this technique, perfusion distal to the injury is prioritized until an in-line flow can be obtained. By utilizing the best features of extracorporeal, endovascular, and open techniques, the outcomes for lower extremity arterial injury can be optimized, such as a reduction in the rate of amputation with quality limb recovery.

## Discussion

Sheath-sheath shunts have been utilized in the past, primarily in patients undergoing complex vascular repair limiting distal perfusion. One series of twelve cases of abdominal aortic aneurysm repair showed a reduction of warm ischemic time by 14 min [7]. Another demonstration of this technique showed restoration of up to 73% of the flow that would otherwise be provided by a Pruitt-Inahara shunt and systolic pressures up to 70 mmHg using 7 French sheaths [8]. Applying these concepts to patients with traumatic popliteal injury may help restore distal flow in both the prehospital and the initial hospital setting, preserving tissue until further management and/or transfer can be performed.

Lower extremity arterial injuries are associated with high amputation rates and poor outcomes due to multifactorial reasons. Revascularization approaches for a popliteal artery injury, such as endovascular placement of a stent across the popliteal artery, are controversial due to knee biomechanics. A medial approach is often utilized for traumatic popliteal artery injury; however, a medial approach increases operative time and the risk of devascularization and necrosis of the gastrocnemius [9, 10]. Alternatively, a posterior approach, as described in the LESR technique, allows for better visualization of the popliteal vessel and can reduce the need for grafting and decrease the length of bypass conduits.

To best address the multifactorial contributions to poor outcomes in popliteal arterial injury, we offer a novel paradigm for traumatic artery injury repair that utilizes innovations in endovascular therapy combined with definitive open reconstruction. The LESR technique may have the potential to decrease patient morbidity and rates of amputation when presenting with traumatic popliteal injury and an associated bony injury requiring fixation.

## Conclusion

We describe the technique of lower extremity staged revascularization, which combines extracorporeal blood shunting, temporary endovascular covered stent placement, external fixation of bony injury, and definitive open repair. We hypothesize that this approach could minimize the factors contributing to poor outcomes after traumatic popliteal artery injury. By using this technique with combined endovascular therapy and definitive open repair, we believe that lower extremity arterial injury outcomes can be improved.

## Data Availability

Not applicable.

## References

[1] Dua A, Desai SS, Shah JO, Lasky RE, Charlton-Ouw KM, Azizzadeh A (2014). Outcome predictors of limb salvage in traumatic popliteal artery injury. Ann Vasc Surg.

[2] Fainzilber G, Roy-Shapira A, Wall MJ, Mattox KL (1995). Predictors of amputation for popliteal artery injuries. Am J Surg.

[3] Hafez HM, Woolgar J, Robbs JV (2001). Lower extremity arterial injury: results of 550 cases and review of risk factors associated with limb loss. J Vasc Surg.

[4] Mullenix PS, Steele SR, Andersen CA, Starnes BW, Salim A, Martin MJ (2006). Limb salvage and outcomes among patients with traumatic popliteal vascular injury: an analysis of the National Trauma Data Bank. J Vasc Surg.

[5] Kröger K, Santosa F, Goyen M (2004). Biomechanical Incompatibility of Popliteal Stent Placement. J Endovasc Ther.

[6] Edwards J, Stonko DP, Abdou H, Treffalls RN, Walker P, Rasmussen TE, et al. Lower extremity extracorporeal distal revascularization (LEEDR) in a swine model of prolonged extremity ischemia. Ann Vasc Surg. 2022:S0890509622006355. 10.1016/j.avsg.2022.09.060. 10.1016/j.avsg.2022.09.060 36441096

[7] Treffalls RN, Stonko DP, Edwards J, Abdou H, Lang E, Walker P, et al. Effects on lower extremity perfusion when the Lower Extremity Extracorporeal Distal Revascularization (LEEDR) system is used for arterial shunting. Protoc Exch. 2022. 10.21203/rs.3.pex-1932/v1.

[8] Österberg K, Falkenberg M, Resch T (2014). Endovascular technique for arterial shunting to prevent intraoperative ischemia. Eur J Vasc Endovasc Surg Off J Eur Soc Vasc Surg.

[9] Makaloski V, Stellmes A, Wyss D, Weiss S, Becker D, Wyss TR (2018). Posterior approach for revascularization in blunt popliteal vessel injury. Ann Vasc Surg.

[10] Zhu Y, Xu Y, Li J, Wang Y, Luo G (2010). Medial approach for popliteal artery injuries. Chin J Traumatol Zhonghua Chuang Shang Za Zhi.

